# Human longevity is associated with regular sleep patterns, maintenance of slow wave sleep, and favorable lipid profile

**DOI:** 10.3389/fnagi.2014.00134

**Published:** 2014-06-24

**Authors:** Diego Robles Mazzotti, Camila Guindalini, Walter André dos Santos Moraes, Monica Levy Andersen, Maysa Seabra Cendoroglo, Luiz Roberto Ramos, Sergio Tufik

**Affiliations:** ^1^Departamento de Psicobiologia, Universidade Federal de São PauloSão Paulo, Brazil; ^2^Disciplina de Geriatria e Gerontologia, Universidade Federal de São PauloSão Paulo, Brazil; ^3^Departamento de Medicina Preventiva, Universidade Federal de São PauloSão Paulo, Brazil

**Keywords:** sleep, longevity, slow wave sleep, actigraphy, lipid profile, EEG spectral analysis

## Abstract

Some individuals are able to successfully reach very old ages, reflecting higher adaptation against age-associated effects. Sleep is one of the processes deeply affected by aging; however few studies evaluating sleep in long-lived individuals (aged over 85) have been reported to date. The aim of this study was to characterize the sleep patterns and biochemical profile of oldest old individuals (*N* = 10, age 85–105 years old) and compare them to young adults (*N* = 15, age 20–30 years old) and older adults (*N* = 13, age 60–70 years old). All subjects underwent full-night polysomnography, 1-week of actigraphic recording and peripheral blood collection. Sleep electroencephalogram spectral analysis was also performed. The oldest old individuals showed lower sleep efficiency and REM sleep when compared to the older adults, while stage N3 percentage and delta power were similar across the groups. Oldest old individuals maintained strictly regular sleep-wake schedules and also presented higher HDL-cholesterol and lower triglyceride levels than older adults. The present study revealed novel data regarding specific sleep patterns and maintenance of slow wave sleep in the oldest old group. Taken together with the favorable lipid profile, these results contribute with evidence to the importance of sleep and lipid metabolism regulation in the maintenance of longevity in humans.

## Introduction

Sleep is essential for physical and mental well-being and is one of the most important factors responsible for the maintenance of a healthy organism, thus representing a homeostatic need required for life (Tufik et al., 2009). Both rapid eye movement (REM) and non-REM (NREM) sleep have been described as having crucial roles in a way that and changes in their quantity and distribution are associated with physical, behavioral, metabolic, and cognitive impairment, in turn linked to increased risk of developing chronic diseases (Levy et al., 2009; Czeisler, 2011; Pack and Pien, 2011; Lal et al., 2012).

It is well known that in humans, sleep goes through an ontogenetic process of alterations that starts in the newborn and lasts for the entire lifespan. With increasing age, the sleep architecture changes in sleep time, proportions of each sleep stage and occurrence of sleep associated events. In general, increase in sleep latency, stages N1 and N2 percentage and wake after sleep onset (WASO), as well as a decrease in total sleep time (TST), sleep efficiency, slow wave sleep and REM sleep have been reported to be associated with aging (Ohayon et al., 2004; Espiritu, 2008). Moreover, reduction in electroencephalogram (EEG) spectral power in NREM and REM sleep and a decrease in delta activity were also reported (Smith et al., 1977; Landolt and Borbely, 2001). In addition to structural changes, older people experience phase advance, a poorly understood phenomena characterized by the tendency to sleep and wake-up earlier (Ancoli-Israel, 1997) and have increased prevalence of sleep disorders, which are significantly associated with morbidity and mortality (Dew et al., 2003; Mazzotti et al., 2012a). The factors underlying sleep disorders in the elderly are complex and a large body of evidence indicates interrelationships between changes in sleep architecture, circadian misalignment, increased risk for medical and psychiatric conditions and medication use (Roepke and Ancoli-Israel, 2010). Indeed, although known as bidirectional, epidemiological evidence is still warranted to better characterize causal relationships between sleep and aging.

Although sleep in older adults has been well studied, few studies have specifically aimed to investigate sleep patterns in individuals over 80 years old. It is believed that individuals who are able to successfully reach very old ages might develop higher adaptation against the age-associated effects; however, the mechanisms involved in this process are still not completely known (Barbieri et al., 2008). Previous reports on sleep in long-lived individuals are mainly epidemiological studies evaluating self-report questionnaires of sleep quality or declared TST (Gu et al., 2010; Chang-Quan et al., 2012). A positive relationship between sleep quality, survival and successful aging has been reported in centenarians, indicating the importance of healthy sleep habits to longevity (Tafaro et al., 2007). Besides, it is known that long-lived individuals present higher total cholesterol, VLDL-cholesterol and triglycerides, and lower HDL-cholesterol levels, which contributes to protection against cardiovascular events and thus extending health- and lifespan (Cullen, 2000). Given the intrinsic relationship between sleep, circadian rhythm, and metabolism (Tevy et al., 2013), we could propose that age-related changes in sleep patterns might be associated to disturbances in lipid metabolism pathways and consequently to reduced lifespan.

Taking into account the lack of comprehensive studies about the quantitative characterization of sleep in individuals that have reached very old age and the need to better understand the role of sleep and its related features in maintaining longevity, the aim of the present study was to characterize the sleep patterns and biochemical profile of long-lived individuals and compare them to the young and older adults to identify possible sleep signatures of longevity in humans.

## Materials and methods

### Ethics statement

The study protocol was approved by the Research Ethics Committee of the Universidade Federal de São Paulo (CEP 1990/08) and all volunteers read and signed the informed consent form, according to the Declaration of Helsinki. This study is also part of a larger study registered in ClinicalTrials.gov (Name: Genetic and Physiological Aspects of Oxidative Profile in Sleep and Well-succeed Aging; Number: NCT01480037; URL: http://clinicaltrials.gov/ct2/show/NCT01480037?term=NCT01480037&rank=1).

### Studied sample and study protocol

One hundred and thirty-two young adult (20–30 years old) and older adult (over 60 years old) males, drawn either from ongoing epidemiological studies in São Paulo, Brazil (Elderly Epidemiological Study) or recruited through local advertising underwent a phone or in person interview about health and lifestyle habits. Only male individuals were selected in order to reduce the effects of female reproductive cycle on sleep (Hachul et al., 2010). After this screening, individuals showing severe chronic disease such as cancer, cardiovascular disease, digestive disorders, type 2 diabetes or neurological and psychiatric antecedents were excluded from the protocol, unless they were under active and stable medical treatment. After selection criteria, 38 individuals were included in the protocol and were distributed into three groups according to their age: (1) Oldest old (*N* = 10, age range 85–105 years old); (2) Older adult (*N* = 13, age range 60–70 years old); and (3) Young adult (*N* = 15, age range 20–30 years old). All participants were invited to spend one night at the Sleep Institute in São Paulo, Brazil, where they were informed about the study protocol, underwent standardized questionnaire data collection, full-night polysomnography (PSG) and blood collection. After up to 23 weeks, the participants of the young adult and older adult groups were contacted again, underwent a second night of PSG, and were invited to use an actigraph and complete a sleep diary for 1 week starting from this second night. Due to limitations associated with their age (the mobility difficulty and the need of a relative to be with them during the PSG), individuals from the oldest old group underwent only one night of PSG. These individuals were also invited to use the actigraph and complete the sleep diary for 1 week after the PSG night.

### Questionnaires

The participants answered the following questionnaires in order to better characterize their sleep quality and patterns: Pittsburgh Sleep Quality Index (PSQI) (Buysse et al., 1989), Insomnia Severity Index (ISI) (Morin et al., 2011), Horne and Ostberg (HO) morningness eveningness questionnaire (Horne and Ostberg, 1976); Epworth Sleepiness Scale (ESS) (Johns, 1991); Katz Index of Independence in Activities of Daily Living (ADL) (Katz et al., 1963); Mini-Mental State Examination (MMSE) (Folstein et al., 1975); and Geriatric Depression Scale (Yesavage et al., 1982). For the young adult and older adult groups, the PSQI and ISI questionnaires were completed on the first visit and all the others on the second visit, except for the ESS, that was applied in both visits. For the oldest old group, all questionnaires were completed on the single visit. Data from the geriatric-specific questionnaires were reported only for the older adult and oldest old groups.

### Polysomnography

Young adult and older adult individuals underwent two non-consecutive full-night PSGs and oldest old individuals underwent a single night of measurements. PSG were performed using the digital system EMBLA® S7000 (Embla Systems Inc., USA). Biological variables were monitored continuously using electroencephalography (F3-M2, F4-M1, C3-M2, C4-M1, and O1-M2 derivations), electrooculography, submental and anterior tibialis electromyography and electrocardiogram (V2 derivation). Airflow was measured using a thermocouple and pressure transducer and respiratory effort was detected using chest and abdominal piezoelectric sensors. Oxygen saturation and pulse were recorded with a pulse oximeter. Sleep scoring and associated events were performed by an experienced sleep technician following the recommended criteria of the American Academy of Sleep Medicine Manual for Scoring Sleep and Associated Events (Iber et al., 2007). The analyzed parameters comprised sleep latency (minutes), REM sleep latency (minutes), TST (minutes), sleep efficiency (%), stage N1 (%), stage N2 (%), stage N3 (%), stage REM sleep (%), minutes awake, arousal index (Arousals/h), periodic limb movement index (PLM/h), apnea-hypopnea index (AHI), baseline oxyhemoglobin (Baseline SO_2_, %), mean oxygen saturation (Mean SO_2_, %) and minimum oxygen saturation (Minimum SO_2_, %).

First night effect of young adult and older adult individuals was verified, since they were subjected to two PSGs. No major differences were found in the sleep parameters between the first and second nights in both groups (Supplementary Tables 1, 2). However, we used the parameters of the first night to compare the PSG parameters among the three groups, without introducing the potential bias of the adaptation night.

### Sleep EEG spectral analysis

The spectral analysis of sleep EEG was performed as previously described (Mazzotti et al., 2012b) with some modifications. In brief, a specific syntax in R (version 2.10.1) was used, in which EEG waves from O2-M1, C4-M1, and F4-M1 derivations were decomposed into delta (<4 Hz), theta (4–7.9 Hz), alpha 1 (8–9.9 Hz), alpha 2 (10–12.9 Hz), beta 1 (13–17.9 Hz), beta 2 (18–29.9 Hz), and gamma (≥30 Hz) frequency bands using the fast Fourier transformation, with sampling rate of 200 Hz and epochs of 30 s. The derivations were chosen based on the visual overall quality of the signal across individuals. The filter settings used were according to standard criteria of sleep EEG data acquisition (low frequency filter of 0.3 Hz; high frequency filter of 35 Hz; time constant of 0.3 s; and notch filter of 60 Hz). The descriptive data [mean, standard deviation (*SD*), median, and interquartile range] from each 30 s window were calculated, and the 5% of time windows with the highest signal amplitude at each sleep stage were considered as outliers and excluded from analysis.

### Actigraphy

To evaluate the rest and activity patterns, wrist activity was recorded using the *Motionlogger Watch* actigraph (Ambulatory Monitoring, Inc., USA) for 1 week, following the second night of PSG for the young adult and older adult groups or after the single night for the oldest old group. Data acquisition was performed using the zero-crossing mode in 1-min epochs according to a previously published validation study (de Souza et al., 2003) and using default settings. A sleep diary was also completed by the individuals to determine and synchronize specific events such as wake-up and sleep schedules, naps, and periods of removal of the actigraph. Sleep (rest) and wake (active) periods were automatically scored using the Cole–Kripke algorithm (Cole et al., 1992) in the Action-W version 2 software (Ambulatory Monitoring, Inc., USA). The following parameters were analyzed: mean and coefficient of variation (CV) of nocturnal sleep and wake-up times and start/end times of diurnal naps, mean and CV of sleep period duration and nap duration, mean sleep efficiency (%), mean sleep latency (minutes), mean WASO (minutes), and number of naps. Analyses were performed for all days of actigraph usage (seven consecutive days) and separately for weekdays and weekends.

### Laboratory measurements

After the second night of PSG (young adult and older adult groups) or the single night (oldest old group), peripheral blood was collected to measure the following levels using standard routine methodologies as indicated in parenthesis: fasting glucose (enzymatic colorimetric), urea (enzymatic colorimetric), creatinine (colorimetric), albumin (colorimetric), total cholesterol (enzymatic colorimetric), LDL-cholesterol (enzymatic colorimetric), HDL-cholesterol (enzymatic colorimetric), VLDL-cholesterol (enzymatic colorimetric), and triglycerides (enzymatic colorimetric). All samples were collected between 06:00 and 08:00 AM while individuals were fasting and biochemical analyses were performed using automated dosages with an Advia® 1650 chemistry system (Siemens Healthcare Diagnostics Inc., USA).

### Statistical analysis

A Shapiro–Wilk test was performed for all continuous variables to verify normality of the data. The questionnaire outcomes, polysomnographic, and actigraphic sleep parameters, as well as the biochemical levels were compared among young adult, older adult and oldest old groups using One-Way analysis of variance (ANOVA) with Bonferroni *post-hoc* test or Kruskal–Wallis test followed by pairwise Mann–Whitney test comparisons. Spectral analysis results were Z-score standardized and the effects of age on frequency specific spectral power at each sleep stage and at each derivation were verified using One-Way ANOVA with Bonferroni *post-hoc* test. All statistical analyses were performed using PASW Statistics 18 (SPSS Inc., USA) and the significance level was set at 0.05. Results are mainly represented by mean ± *SD*.

## Results

### Sample characteristics

The studied sample consisted of male individuals from three distinct age groups. Mean ± *SD* age for young adult, older adult, and oldest old individuals was 24.33 ± 2.19, 65.54 ± 3.05, and 91.9 ± 6.06 years, respectively. Body mass index was 24.04 ± 2.74 for young adult, 25.87 ± 3.56 for older adult, and 24.96 ± 3.55 for oldest old, and did not statistically differ among groups [*F*_(2, 35)_ = 1.103; *p* = 0.343]. Six individuals from the older adult group and five from the oldest old group showed controlled chronic diseases (type 2 diabetes, dyslipidemia or hypertension) and were taking medications at the time of the PSG.

### Subjective sleep quality and pattern measured by questionnaire assessment

To assess subjective sleep quality and verify the effect of aging and longevity on these patterns a variety of validated questionnaires were applied in the three studied groups. No significant differences were found for the outcomes of the following questionnaires among young adult, older adult and oldest old individuals, respectively: PSQI (5.2 ± 2.1, 4.1 ± 1.7, and 7.0 ± 4.6, Kruskal–Wallis test χ^2^ = 4.308, *p* = 0.116), ISI (4.3 ± 3.3, 2.4 ± 1.9, and 5.6 ± 5.0, Kruskal–Wallis test χ^2^ = 4.907, *p* = 0.086), ESS applied in the first visit [8.3 ± 4.6, 6.3 ± 3.8, and 8.8 ± 4.1, ANOVA *F*_(2, 33)_ = 1.131, *p* = 0.335] and ESS applied in the second visit (young adult and older adult only, respectively: 7.6 ± 4.3 and 6.4 ± 3.8, Mann–Whitney *U* = 60.00, *p* = 0.485). Nevertheless, the HO morningness eveningness questionnaire revealed significant differences among the groups [ANOVA *F*_(2, 28)_ = 21.444, *p* < 0.001]. Both older adults (67.9 ± 12.8) and oldest old (66.8 ± 9.0) individuals showed higher scores than young adults (42.2 ± 9.7, *post-hoc* test for both comparisons *p* < 0.001) indicating that the aging is associated with morning chronotype while young individuals tend to have an evening chronotype. No significant difference between older adult and oldest old groups was found for this outcome.

Geriatric-specific questionnaires were also applied for the older adult and oldest old groups in order to better evaluate their status. Significant differences were found between groups for the Katz Index of Independence in ADL (Mann–Whitney *U* = 45.4, *p* = 0.039). More specifically, three individuals from the oldest old group presented at least one degree of dependence and only one presented dependence in three activities. Moreover, oldest old individuals showed significantly lower MMSE scores (22.8 ± 5.6) and higher GDS scores (2.8 ± 2.1) than older adults (28.2 ± 1.1, Mann–Whitney *U* = 9.5, *p* < 0.001, and 0.9 ± 1.4, Mann–Whitney *U* = 23.0, *p* = 0.037, respectively). These results indicate a slight impairment in cognitive, physical and behavioral aspects specific to older ages that should be taken into account when investigating the parameters of sleep quality and quantity in these individuals. However, differences in geriatric-specific questionnaires did not modify the association results described in the following sections (data not shown).

### Polysomnographic parameters and sleep EEG spectral power

The PSG parameters used to verify the effect of aging and longevity were derived from the first PSG night for all individuals. Table 1 shows the results for young adult, older adult, and oldest old individuals. In summary, oldest old individuals showed significantly higher REM sleep latency, lower TST, lower sleep efficiency, lower REM sleep and more minutes awake than young adult and older adult (*p* < 0.05, Table 1). In addition, the older adult and the oldest old showed more arousals/h, more PLM/h, higher AHI and lower baseline and minimum SO_2_ than young adult (*p* < 0.05, Table 1). The older adult also showed significantly lower TST, sleep efficiency and mean SO_2_ than young adult individuals (*p* < 0.05, Table 1). No significant differences in sleep latency or stage N2 were found for any of the comparisons. Although a slight decrease in stage N3 was observed both in older adult (20.6 ± 6.6) and oldest old individuals (19.1 ± 17.5) when compared to young adult (25.7 ± 5.6; *p* = 0.287), no trend toward significant decrease was detected when older adult and oldest old subjects were compared. The present findings on the macrostructure of sleep indicate that overall, oldest old individuals showed decreased sleep quality parameters compared to the other groups, but maintained some specific parameters such as stage N3.

**Table 1:** **Polysomnographic parameters measured in young adults, older adults, and oldest old individuals**.

**Polysomnographic parameter**	**YA (*N* = 15)**	**OA (*N* = 13)**	**OO (*N* = 10)**	***F*_(2, 35)_ or χ^2b^**	***p***	***Post-hoc p*-value**		
	**Mean (*SD*)**	**Mean (*SD*)**	**Mean (*SD*)**			**YA × OA**	**YA × OO**	**OA × OO**
Sleep latency (min)	20.4 (23.5)	13.5 (11.4)	41.4 (43.9)	4.004	0.135		NS	
REM sleep latency (min)^a^	96.5 (28.3)	100.8 (49.0)	182.0 (102.3)	7.229	0.027	0.662	0.006	0.043
Total sleep time (min)	383.0 (29.5)	343.8 (55.9)	198.6 (90.5)	21.522	<0.001	0.048	<0.001	0.001
Sleep efficiency (%)	89.4 (6.0)	77.4 (12.7)	45.7 (19.4)	23.377	<0.001	0.008	<0.001	0.001
Stage N1 %	7.6 (2.6)	16.7 (7.3)	27.5 (20.1)	18.889	<0.001	<0.001	0.002	0.163
Stage N2 %	47.3 (7.6)	42.7 (7.9)	43.5 (9.2)	1.292	0.287		NS	
Stage N3 %	25.7 (5.6)	20.6 (6.6)	19.1 (17.5)	1.472	0.243		NS	
Stage REM %	19.5 (4.2)	20.0 (5.7)	9.9 (8.8)	9.092	0.011	0.534	0.007	0.011
Minutes awake	29.5 (15.0)	88.5 (56.7)	172.5 (66.1)	23.173	<0.001	0.001	<0.001	0.008
Arousals/h	8.8 (2.8)	23.8 (11.2)	31.6 (26.1)	17.863	<0.001	<0.001	0.002	0.804
Periodic limb movements/h	0.1 (0.3)	12.8 (23.5)	14.6 (14.2)	12.804	0.002	0.002	0.001	0.466
Apnea-hypopnea index	1.7 (3.1)	16.7 (17.2)	30.3 (29.0)	20.075	<0.001	<0.001	<0.001	0.215
Baseline SO_2_ (%)	97.0 (0.9)	94.4 (1.7)	95.0 (2.8)	13.308	0.001	<0.001	0.040	0.264
Mean SO_2_ (%)	96.3 (1.0)	94.1 (1.8)	94.2 (2.8)	8.728	0.013	0.002	0.107	0.804
Minimum SO_2_ (%)	92.8 (1.9)	87.0 (5.6)	88.0 (6.0)	9.737	0.008	0.007	0.011	0.513

REM, rapid eye movement; min, minutes; h, hour; SO_2_, oxyhemoglobin saturation; SD, standard deviation; N, number of individuals; NS, non-significant; YA, young adult; OA, older adult; OO, oldest old;

a Two individuals from the oldest old group did not reach REM sleep, so their REM sleep latency were considered as missing values and their Stage REM % were zero;

b *One-Way analysis of variance (Stage N2 % and Stage N3 %) followed by Bonferroni post-hoc test or Kruskal–Wallis test followed by pairwise Mann–Whitney tests (other variables)*.

**Figure 1:**
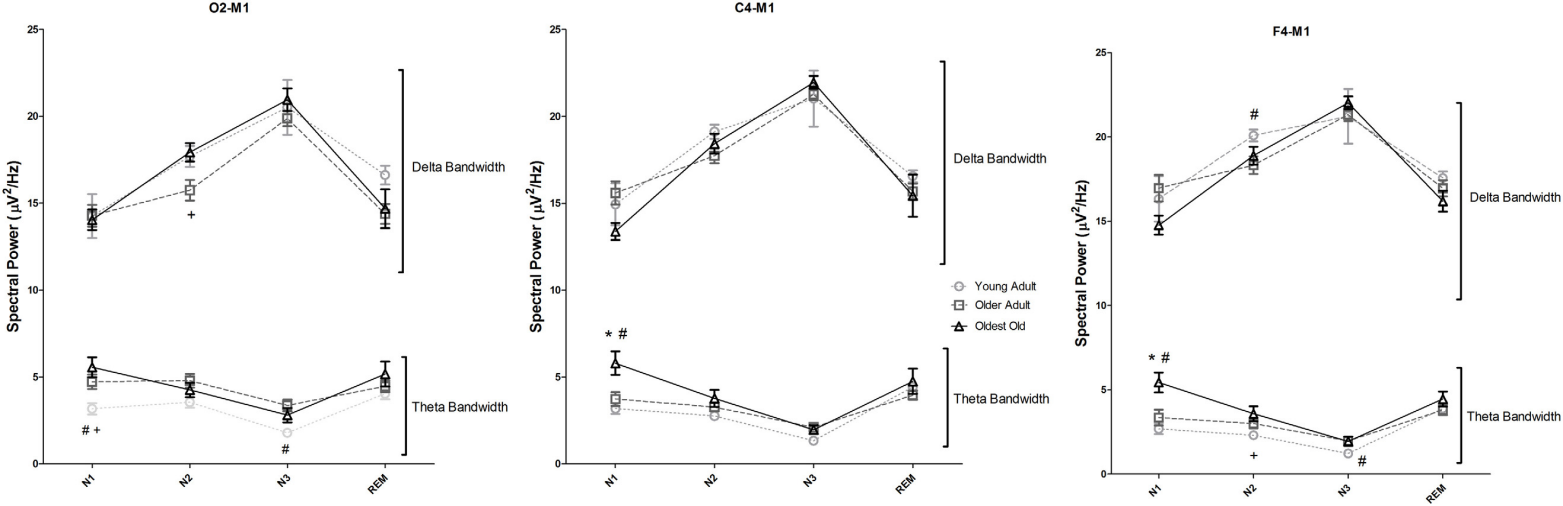
**Sleep electroencephalogram spectral analysis recorded using O2-M1, C4-M1, and F4-M1 derivations**. Spectral power is shown for Delta (<4 Hz) and Theta (4–7.9 Hz). One-Way analysis of variance followed by Bonferroni *post-hoc* test using the Z-score of the spectral power as dependent variables. ^*^p < 0.05 vs. Young Adult; ^#^*p* < 0.05 vs. older adult; ^+^*p* < 0.05 vs. oldest old. Error bars represent standard error of the mean.

The spectral analysis of sleep EEG was also performed using data derived from the first PSG night. Figure 1 represents the delta and theta spectral power for each sleep stage separately (N1, N2, N3, and REM), recorded by the selected EEG derivations. The full results of the spectral power in all frequency bands, sleep stages and derivations are shown in Supplementary Table 3 and in Supplementary Figure 1. Overall, oldest old individuals showed higher theta power than young adults and older adults in stage N1, verified both in C4-M1 (*p* = 0.001 and *p* = 0.012, respectively) and F4-M1 derivations (*p* = 0.001 and *p* = 0.010, respectively). In addition, older adults showed lower delta power in stage N2 than young adults (F4-M1, *p* = 0.030), and oldest old individuals (O2-M1, *p* = 0.043). Theta power in young adults was significantly lower than in oldest old individuals in stage N2 (F4-M1, *p* = 0.016) and lower than older adults in stage N3 (F4-M1, *p* = 0.036). Interestingly, no significant differences in stage N3 delta power among groups in all studied derivations were found (*p* > 0.05).

Fast EEG waves (alpha 1, alpha 2, beta 1, and beta 2) also showed specific patterns in longevity (Supplementary Table 3 and Supplementary Figure 1). While older adults showed overall significantly higher power of these fast frequency bands than young adults, these differences were not statistically significant in the oldest old group when compared to young adults, suggesting similarity of patterns between these groups.

### Rest and activity patterns in aging and longevity

The rest and activity patterns were characterized for the studied individuals using wrist actigraphy. The effect of age on investigated parameters represented by their mean or CV on seven consecutive days of actigraph usage is shown on Table 2. In summary, oldest old individuals slept earlier, showed lower mean sleep efficiency and more time WASO than young adults and older adults. Moreover, oldest old individuals woke up earlier, showed less variation in sleep onset time, wake-up time and sleep duration than young adults, indicating a higher degree of regularity in sleep schedules. Although older adults also showed less variation in the same parameters when compared to young adults, these differences were not significant.

**Table 2:** **Measured actigraphic parameters among young adult, older adult, and oldest old groups**.

**Actigraphic parameter**	**YA (*N* = 11)**	**OA (*N* = 7)**	**OO (*N* = 7)**	***F*_(2, 22)_ or χ^2a^**	***p***	***Post-hocp*-value**		
	**Mean (*SD*)**	**Mean (*SD*)**	**Mean (*SD*)**			**YA × OA**	**YA × OO**	**OA × OO**
Mean sleep time (hh:mm)	1:06 AM (01:13)	12:14 AM (00:51)	9:57 PM (1:05)	17.639	<0.001	0.365	<0.001	0.002
Sleep time CV (%)	6.4% (2.9%)	3.5% (1.2%)	3.0% (1.9%)	8.427	0.015	0.023	0.014	0.404
Mean wake-up time (hh:mm)	8:52 AM (01:24)	7:36 AM (00:34)	6:55 AM (01:23)	5.758	0.010	0.139	0.011	0.918
Wake-up time CV (%)	21.0% (8.2%)	13.4% (5.2%)	6.9% (4.3%)	10.077	0.001	0.079	0.001	0.395
Mean sleep duration (min)	467.3 (84.7)	442.9 (62.3)	538.4 (59.0)	3.351	0.054		NS	
Sleep duration CV (%)	21.4% (6.9%)	14.6% (6.7%)	9.2% (5.3%)	7.948	0.003	0.118	0.002	0.395
Mean sleep efficiency (%)	96.9 (1.8)	96.8 (1.7)	89.7 (4.3)	13.695	0.001	0.751	0.001	0.003
Mean sleep latency (min)	10.8 (5.6)	9.6 (5.0)	11.8 (4.5)	1.401	0.496		NS	
Mean WASO (min)	14.9 (10.2)	14.0 (6.5)	52.6 (15.2)	14.086	0.001	0.994	0.001	0.002

*These parameters represent the mean or coefficient of variation of seven consecutive days of actigraph usage*.

N, number of individuals; CV, coefficient of variation; min, minutes; SD, standard deviation; NS, non-significant; WASO, wake after sleep onset; YA, young adult; OA, older adult; OO, oldest old;

a *One-Way analysis of variance followed by Bonferroni post-hoc test (Mean Sleep Time, Mean Wake-up Time, Wake-up time CV, and sleep duration CV) or Kruskal–Wallis test followed by pairwise Mann–Whitney tests (other variables)*.

The number of naps reported by the participants and verified by the actigraph recordings was also measured and differed among groups (Kruskal–Wallis *X*^2^ = 6.696, *p* = 0.031). Oldest old individuals also had significantly more naps (3.71 ± 2.36) than young adults (0.73 ± 0.79; *p* = 0.015). Nap start and end times, duration and their respective CV did not differ between older adult and oldest old (*p* > 0.05, Supplementary Table 4). For all actigraphic parameters, no major differences were found when weekdays and weekends were analyzed separately (Supplementary Table 4).

### Biochemical measurements in aging and longevity

Table 3 represents the biochemical levels of the studied groups. Overall, older adults, but not the oldest old, showed higher triglyceride (*p* = 0.009), total cholesterol (*p* = 0.030), and VLDL-cholesterol levels (*p* = 0.010) than young adults. Furthermore, HDL-cholesterol levels in the oldest old group were higher than both young (*p* = 0.032) and older adults (*p* = 0.020). Additionally, young adults showed lower glucose levels than older adults (*p* = 0.004) and oldest old individuals (*p* = 0.003) and higher albumin levels than oldest old individuals (*p* = 0.007). Taken together, these findings suggest that the oldest old individuals present a more favorable lipid profile than older adults.

**Table 3:** **Biochemical levels of young adults, older adults, and oldest old individuals**.

**Biochemical levels**	**YA (*N* = 15)**	**OA (*N* = 13)**	**OO (*N* = 10)**	***F*_(2, 35)_ or χ^2a^**	***p***	***Post-hoc p*-value**		
	**Mean (*SD*)**	**Mean (*SD*)**	**Mean (*SD*)**			**YA × OA**	**YA × OO**	**OA × OO**
Fasting glucose (mg/dL)	87.3 (6.6)	103.5 (21.1)	100.0 (10.4)	11.62	0.003	0.004	0.003	0.877
Urea (mg/dL)	34.3 (6.8)	36.1 (10.4)	47.6 (42.3)	0.649	0.723		NS	
Creatinine (mg/dL)	1.1 (0.1)	1.1 (0.1)	1.4 (0.7)	4.059	0.131		NS	
Albumin (g/dL)	4.7 (0.2)	4.6 (0.3)	4.3 (0.3)	5.487	0.009	0.654	0.007	0.129
Total cholesterol (mg/dL)	159.1 (25.8)	185.4 (25.2)	181.7 (25.3)	4.337	0.021	0.030	0.109	1.000
HDL-cholesterol (mg/dL)	45.9 (8.8)	46.2 (10.4)	54.0 (8.6)	6.459	0.040	0.782	0.032	0.020
LDL-cholesterol (mg/dL)	95.4 (22.9)	112.8 (22.5)	108.6 (22.5)	2.232	0.122		NS	
VLDL-cholesterol (mg/dL)	17.7 (6.5)	26.4 (9.5)	19.1 (4.6)	7.623	0.022	0.010	0.344	0.058
Triglycerides (mg/dL)	88.7 (32.2)	131.6 (47.8)	95.3 (22.6)	7.691	0.021	0.009	0.360	0.063

SD, standard deviation; NS, non-significant; YA, young adult; OA, older adult; OO, oldest old;

a *One-Way analysis of variance with Bonferroni post-hoc test (albumin, total cholesterol, and LDL-cholesterol); Kruskal–Wallis followed by pairwise Mann–Whitney tests (other variables)*.

## Discussion

The characterization of the physiological aspects of sleep in oldest old individuals has brought some new insights to the understanding of the role of sleep in maintaining the functioning organism in very old age. The main findings of the present study were that, although oldest old individuals showed poorer sleep quality, no significant differences in the percentage of stage N3 or in stage N3 delta power were detected when compared to the older adult group. In addition, the actigraphic measurements revealed that oldest old individuals maintain strictly regular sleep and wake schedules and laboratory examinations showed that these individuals also presented a better lipid profile than older adults in our study.

Alterations in sleep architecture are expected with aging and might be related to a reduction in the ability to sleep, rather than a decrease in the need for sleep itself (Cooke and Ancoli-Israel, 2006). Our findings corroborate the decrease in TST and sleep efficiency, and higher number of arousals, PLM, and AHI found in previous studies in elderly (Bliwise, 1993). Hoch et al. (1990) focused on the characterization of sleep-disordered breathing in the elderly in their seventh, eighth, and ninth decades of life and found that this disorder increases with advancing age even in the healthy elderly (Hoch et al., 1990). Thus, the duration and efficiency of sleep might be impaired by the higher number of arousals as a consequence of apnea/hypopnea or movement disorder events. In the oldest old individuals, we found that this process was emphasized, which clearly accentuates the impairment observed in the sleep of the very old, as evidenced by the decrease in REM sleep percentage, increase in superficial sleep stages and decline of cognitive and physical aspects as verified by the MMSE and Katz Index of Independence in ADL scores.

Wauquier et al. (1992) also described the sleep structure in healthy elderly males and females with ages ranging from 88 to 102 years and their main findings were the gender differences in sleep parameters. For instance, males presented lower TST, shorter REM sleep latency, and less slow wave sleep than females (Wauquier et al., 1992), but no comparison with younger groups was reported. In previous studies, reduction in slow wave sleep was also characterized in the aged brain (Cirelli, 2012); however, the slight decrease we found in stage N3 percentage was not significant for either older adults or oldest old individuals. On the other hand, no trend toward a decrease in stage N3 when oldest old was compared to older adult was found and no differences in stage N3 delta power were detected either. Therefore, both aging and longevity might have an effect on specific aspects of sleep EEG spectral power. Collectively with the sleep macrostructure results, it seems that the maintenance of slow wave sleep in both quantity (stage N3%) and quality (stage N3 delta power) might be essential in all stages of the human lifespan and might not be subjected to decrease in older ages, relative to early elderly.

A meta-analysis representing more than 3500 individuals across the entire age-spectrum (5–102 years old) found a significant increase in stages N1 and N2 with aging. In the study, when only elderly individuals were investigated, the slow wave sleep amount did not decrease with increasing aging, agreeing with our findings (Ohayon et al., 2004). In addition, sleep homeostasis response to naps, verified by sleep EEG delta power, was reported to behave equally in young and elderly (Campbell and Feinberg, 2005). With these results, we suggest that even in older ages, individuals might have a minimum of slow wave sleep to maintain all their physiological processes and still continue to produce adequate responses to sleep homeostasis, which propose the importance of this stage to successful aging and longevity.

Regarding the other frequency bands, our results have shown specific patterns of theta waves in stage N1 as well as alpha and beta waves mainly in stages N3 and REM in the oldest old individuals, a potential indicative of specific sleep EEG signature in longevity. Cortical activation is directly related to increase in power of high frequency bands in EEG, and previous studies showed that increasing age was associated with higher power in the beta range (Carrier et al., 2001). Interestingly, in our study, although these patterns were observed in older adults, the same was not found in the oldest old group, suggesting that the brain of the oldest old may not be subjected to as much cortical activation as the older adult, despite the increase in the number of arousals (Table 1). In this sense, this evidence may indicate that the older brain might respond differently to cortical activation during sleep; however, longitudinal and neuroimaging studies need to be performed in order to confirm the neurodevelopmental changes that lead to the EEG patterns identified in the oldest old individuals by the present study.

Another interesting feature observed was the phase advance phenomenon, which was clearly characterized in the oldest old individuals, both by the HO morningness and eveningness questionnaire and by the actigraphic recordings. This event has been described in the literature and although it is poorly understood, it is thought to be associated with the inability to maintain sleep at specific circadian phases or as a consequence of alterations in the circadian regulation of physiological processes with aging (Edwards et al., 2010). Nevertheless, oldest old individuals showed strictly regular sleep patterns as evidenced by sleep diary and actigraphic recordings. These individuals tend to go to bed, wake-up, and take naps almost at the same circadian time. These findings did not agree with the decrease of circadian regulation previously mentioned and therefore it might be hypothesized that oldest old individuals are under strict circadian regulation, unlike the older adults. It has been described in Japanese from Okinawa, individuals classified as having good sleep health, that regular sleep schedules and afternoon naps are associated with healthy lifestyle and emotional adaptability (Taira et al., 2002), which are factors that might be associated with survival and ability to reach extreme ages.

In addition, we observed the interesting co-occurrence of the maintenance of slow wave sleep, regular sleep patterns and a favorable lipid profile in the oldest old. It is known that HDL has antioxidant proprieties, and higher HDL-cholesterol levels are associated with antiatherogenic effects (Kontush and Chapman, 2006; Boes et al., 2009). Furthermore, higher total cholesterol, VLDL-cholesterol and triglycerides, and lower HDL-cholesterol levels are important predictors of cardiovascular events (Cullen, 2000), which might reflect a decrease in the lipid metabolism control usually present in the elderly (Mazzotti et al., 2011) and increased mortality. These findings may indicate that oldest old individuals might be considered as survivors, protected against cardiovascular events. Therefore, the well characterized relationship between sleep, circadian rhythm and metabolism (Tevy et al., 2013) suggests that the maintenance of regular sleep patterns and slow wave sleep may be associated to protection against changes in lipid metabolism pathways as a consequence of aging, thus contributing to increased lifespan.

Interestingly, experimental and epidemiological evidence suggests that impairment in slow wave sleep is responsible for a lack of hormonal regulation and has been associated as a risk factor for metabolic syndrome features such as type 2 diabetes and obesity (Van Cauter et al., 2008). In this sense, the maintenance of slow wave sleep in the oldest old identified in this study may be related to a better regulation of lipid metabolism, appetite and hormonal control, reflected by the presence of favorable lipid profile in these individuals. Furthermore, it can be suggested that the regularity in sleep patterns might help to synchronize adequate hormone release and, together with a healthier diet usually present in long-lived individuals, contribute to lower cardiovascular risk and increased longevity. Corroborating with this hypothesis, caloric restriction, one of the most robust preventers or delayers of age-related processes and responsible for significantly extended lifespan (Barzilai et al., 2012), has been previously associated with sleep. In *Drosophila*, high calorie diet, besides reducing lifespan, accelerated age-related process, including sleep changes (Yamazaki et al., 2012). These data support the idea that sleep is strongly regulated by calorie intake, and modulation of this process can modify the longevity in model organisms. To the best of our knowledge, this is the first report characterizing regular sleep patterns in long-lived individuals by using actigraphic recordings. Our findings suggest that the higher degree of regularity in life might be one of the factors related to the healthier lifestyle, diet, and sleep quality of very old individuals. This issue has been discussed before in which not only regularity but also attitude and even religiosity appear as important factors for healthy aging (Walker, 1996). Therefore, to confirm our hypothesis, longitudinal studies focused on regularity in lifestyle, including sleep, are essential and will certainly give us insights into the biology of longevity.

The relationship between sleep and aging has been extensively studied. A number of age-related factors such as inflammation, increased oxidative stress, mitochondrial decline and cellular senescence have been recently described to be affected by sleep and sleep deprivation (Singh et al., 2008; Naidoo, 2009; Rosa Neto et al., 2010; Pellegrino et al., 2012; Anafi et al., 2013). In this sense, we could understand aging as a process composed of recursive and positive feedback events that interact to define the aging phenotype. This complex model makes difficult to establish causal relationships among these events (Watt, 2014). Nevertheless, we could suggest that disrupted sleep-wake cycle and chronic sleep restriction, highly prevalent conditions in the modern society, are strongly associated to age-related diseases. Therefore, the practice of sleep medicine as a tool to contribute with the healthy aging population is essential.

In conclusion, three major features were associated with longevity in this study: the maintenance of slow wave sleep in oldest old individuals when compared to older adults, the existence of strictly regular sleep patterns among oldest old individuals and the occurrence of a favorable lipid profile in these individuals. In addition, signatures of sleep EEG in the brain of oldest old individuals were also described. These findings support the role of sleep and lipid metabolism control in the maintenance of longevity in humans.

### Conflict of interest statement

The authors declare that the research was conducted in the absence of any commercial or financial relationships that could be construed as a potential conflict of interest.

## Supplementary material

Supplementary Table 1 **Evaluation of the first-night effect on the polysomnographic parameters of young adult and older adult individuals**.

Supplementary Table 2 **Spearman correlation matrix of polysomnography parameters between first and second night for young adult and older adult individuals**.

Supplementary Table 3 **Sleep eletroencephalogram spectral power for all studied frequency bands and all derivations and sleep stages**.

Supplementary Table 4 **Actigraphic parameters among young adult, older adult, and oldest old individuals shown separately for weekdays and weekends**. Nap parameters are also shown for older adult and oldest adult individuals.

Supplementary Figure 1 **Sleep electroencephalogram spectral analysis recorded using O2-M1 (A–D), C4-M1 (E–H), and F4-M1 (I–L) derivations**. Spectral power is represented for each frequency band as follows: Delta (<4 Hz), Theta (4–7.9 Hz), Alpha 1 (8–9.9 Hz), Alpha 2 (10–12.9 Hz), Beta 1 (13–17.9 Hz), Beta 2 (18–29.9 Hz), and Gamma (≥30 Hz). One-Way analysis of variance followed by Bonferroni *post-hoc* test using the Z-score standardized spectral power as dependent variables. ^*^*p* < 0.05 vs. young adult; ^#^*p* < 0.05 vs. older adult.
