# Supplementary material for: Human longevity is associated with regular sleep patterns, maintenance of slow wave sleep, and favorable lipid profile
Source: Front Aging Neurosci. 2014 Jun 24;6:134. doi: 10.3389/fnagi.2014.00134 (PMC4067693; doi:10.3389/fnagi.2014.00134)
Supplement: Supplementary Table 1 — Evaluation of the first-night effect on the polysomnographic parameters of young adult and older adult individuals. [file DataSheet1.DOCX]

Supplementary Table 1: Evaluation of the first-night effect on the polysomnographic parameters of young adult and older adult individuals.

|  | Young adult (N=15) | | | | | | |  |  |  | Older adult (N=13) | | | | | | |  |  |
| --- | --- | --- | --- | --- | --- | --- | --- | --- | --- | --- | --- | --- | --- | --- | --- | --- | --- | --- | --- |
| PSG parameter | First PSG night | | |  | Second PSG Night | | | Z^a^ | p |  | First PSG night | | |  | Second PSG Night | | | Z^a^ | p |
|  | Mean | SD | Median |  | Mean | SD | Median |  |  |  | Mean | SD | Median |  | Mean | SD | Median |  |  |
| Sleep Latency (min) | 20.4 | 23.51 | 12.8 |  | 19.1 | 35.9 | 10.7 | -0.937 | 0.349 |  | 13.5 | 11.4 | 10.50 |  | 9.9 | 8.3 | 7.2 | -1.399 | 0.162 |
| REM Sleep Latency (min)^a^ | 96.5 | 28.30 | 85 |  | 87.8 | 43.7 | 82 | -0.824 | 0.410 |  | 100.8 | 49.0 | 91.00 |  | 73.8 | 41.2 | 75.5 | -2.062 | 0.039 |
| Total Sleep Time (min) | 383.0 | 29.47 | 383.5 |  | 361.6 | 74.2 | 386.5 | -1.130 | 0.258 |  | 343.8 | 55.9 | 340.50 |  | 365.5 | 56.8 | 387 | -0.943 | 0.345 |
| Sleep Efficiency (%) | 89.4 | 6.02 | 91.7 |  | 88.5 | 12.2 | 90.6 | -0.454 | 0.649 |  | 77.4 | 12.7 | 78.10 |  | 83.1 | 9.4 | 86.2 | -1.503 | 0.133 |
| Stage N1 % | 7.6 | 2.56 | 7.2 |  | 7.4 | 2.5 | 7.3 | -0.710 | 0.478 |  | 16.7 | 7.3 | 15.40 |  | 14.6 | 7.3 | 11.9 | -1.530 | 0.126 |
| Stage N2 % | 47.3 | 7.56 | 46.9 |  | 45.2 | 7.9 | 44.7 | -0.909 | 0.363 |  | 42.7 | 7.9 | 42.60 |  | 39.8 | 6.6 | 40 | -1.503 | 0.133 |
| Stage N3 % | 25.7 | 5.55 | 23.7 |  | 27.5 | 5.0 | 27.4 | -1.306 | 0.191 |  | 20.6 | 6.6 | 22.90 |  | 24.6 | 5.7 | 23.3 | -1.573 | 0.116 |
| Stage REM % | 19.5 | 4.24 | 18.7 |  | 19.9 | 6.5 | 21.5 | -0.085 | 0.932 |  | 20.0 | 5.7 | 23.00 |  | 21.0 | 4.2 | 20.8 | -0.510 | 0.610 |
| Minutes Awake | 29.5 | 14.99 | 29 |  | 25.5 | 15.8 | 20.8 | -1.193 | 0.233 |  | 88.5 | 56.7 | 81.40 |  | 64.0 | 37.4 | 56.2 | -1.503 | 0.133 |
| Arousals/h | 8.8 | 2.80 | 8.6 |  | 9.3 | 3.6 | 8.5 | -0.483 | 0.629 |  | 23.8 | 11.2 | 22.40 |  | 20.0 | 8.6 | 20.2 | -1.678 | 0.093 |
| PLM/h | 0.1 | 0.34 | 0 |  | 0.5 | 1.2 | 0 | -1.461 | 0.144 |  | 12.8 | 23.5 | 0.60 |  | 14.2 | 26.0 | 1.1 | -0.237 | 0.813 |
| Apnea-Hypopnea Index | 1.7 | 3.12 | 0.7 |  | 1.7 | 2.6 | 0.6 | -0.919 | 0.358 |  | 16.7 | 17.2 | 14.80 |  | 15.9 | 15.4 | 11.9 | -0.524 | 0.600 |
| Baseline SO_2_ (%) | 97.0 | 0.90 | 97.1 |  | 96.9 | 0.9 | 96.85 | -0.105 | 0.916 |  | 94.4 | 1.7 | 94.50 |  | 94.6 | 1.7 | 94.7 | -1.069 | 0.285 |
| Mean SO_2_ (%) | 96.3 | 1.02 | 96.3 |  | 96.2 | 1.0 | 96.3 | -0.350 | 0.726 |  | 94.1 | 1.8 | 93.80 |  | 94.3 | 2.1 | 94.8 | -1.099 | 0.272 |
| Minimum SO_2_ (%) | 92.8 | 1.93 | 93 |  | 92.6 | 2.0 | 92 | -0.454 | 0.650 |  | 87.0 | 5.6 | 87.00 |  | 87.3 | 4.0 | 87 | -0.070 | 0.944 |

REM: rapid eye movement; min: minutes; h: hour; PLM: periodic limb movement; SO_2_: oxyhemoglobin saturation; SD: standard deviation; N: number of individuals; PSG: polysomnography;

^a^: Wilcoxon signed-rank test.
