# Supplementary material for: Human longevity is associated with regular sleep patterns, maintenance of slow wave sleep, and favorable lipid profile
Source: Front Aging Neurosci. 2014 Jun 24;6:134. doi: 10.3389/fnagi.2014.00134 (PMC4067693; doi:10.3389/fnagi.2014.00134)
Supplement: Supplementary Figure 1 — Sleep electroencephalogram spectral analysis recorded using O2-M1 (A–D), C4-M1 (E–H), and F4-M1 (I–L) derivations. Spectral power is represented for each frequency band as follows: Delta (<4 Hz), Theta (4–7.9 Hz), Alpha 1 (8–9.9 Hz), Alpha 2 (10–12.9 Hz), Beta 1 (13–17.9 Hz), Beta 2 (18–29.9 Hz), and Gamma (≥30 Hz). One-Way analysis of variance followed by Bonferroni post-hoc test using the Z-score standardized spectral power as dependent variables. *p < 0.05 vs. young adult; #p < 0.05 vs. older adult. [file DataSheet5.DOCX]

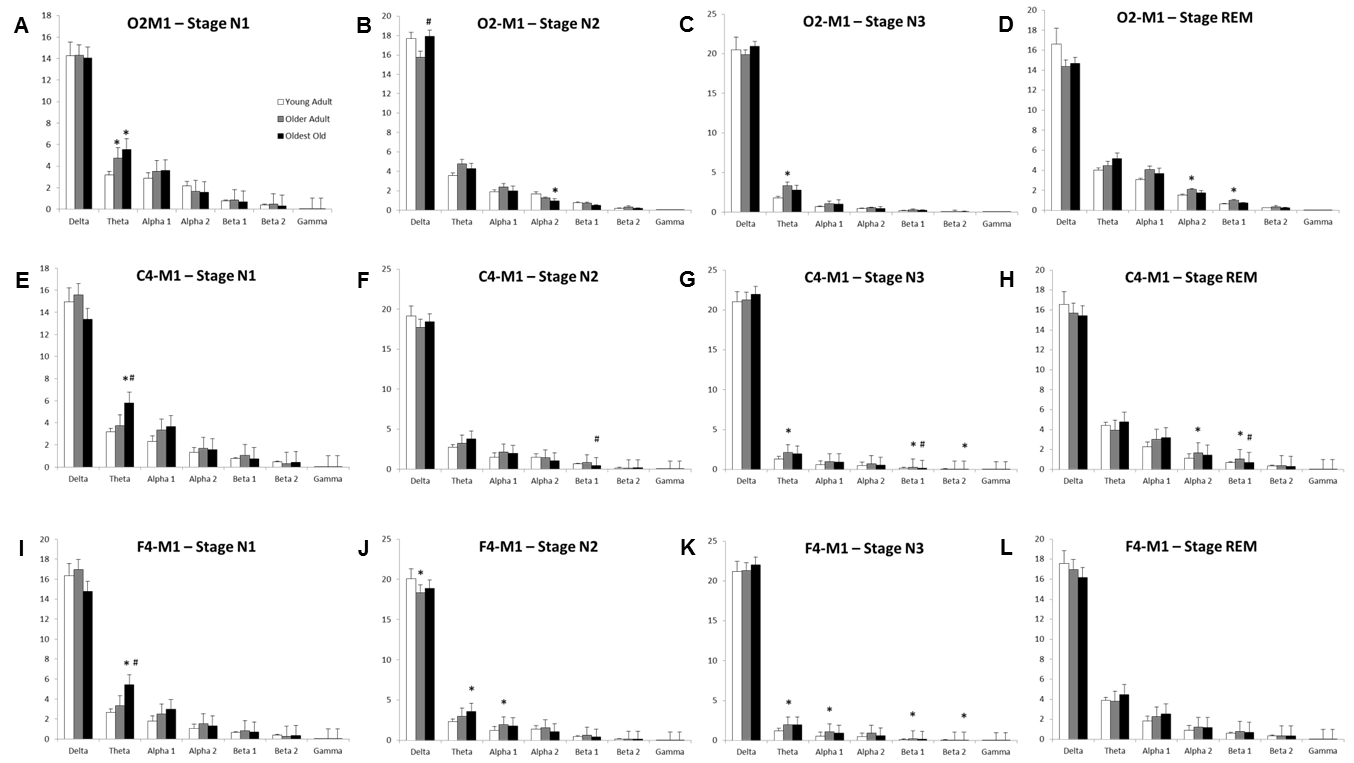


Supplementary Figure 1: Sleep electroencephalogram spectral analysis recorded using O2-M1 (A-D), C4-M1 (E-H) and F4-M1 (I-L) derivations. Spectral power is represented for each frequency band as follows: Delta (<4 Hz), Theta (4–7.9 Hz), Alpha 1 (8–9.9 Hz), Alpha 2 (10–12.9 Hz), Beta 1 (13–17.9 Hz), Beta 2 (18–29.9 Hz) and Gamma (≥30 Hz). One-way analysis of variance followed by Bonferroni *post hoc* test using the Z-score standardized spectral power as dependent variables. *p<0.05 versus young adult; # p<0.05 versus older adult.
